# Initial Study of Feedstock Material Compositions for 3D Printing of Hybrid Metal–Polymer Components via Electrodeposition and Photopolymerization in an Electroplating Bath Environment

**DOI:** 10.3390/molecules31081316

**Published:** 2026-04-17

**Authors:** Dawid Kiesiewicz, Karolina Syrek, Paweł Niezgoda, Szymon Żydowski, Sylwia Łagan, Maciej Pilch

**Affiliations:** 1 Faculty of Civil Engineering, Cracow University of Technology, Warszawska 24, 31-155 Cracow, Poland; 2 Faculty of Chemistry, Jagiellonian University, Gronostajowa 2, 30-387 Cracow, Poland; syrek@chemia.uj.edu.pl; 3 Faculty of Chemical Engineering and Technology, Cracow University of Technology, Warszawska 24, 31-155 Cracow, Poland; 4 Faculty of Mechanical Engineering, Cracow University of Technology, Warszawska 24, 31-155 Cracow, Poland; sylwia.lagan@pk.edu.pl

**Keywords:** electrodeposition, photopolymerization, hybrid components, 3D printing

## Abstract

Hybrid metal–polymer components are used in many industries, such as in aerospace, automotives, and electronics, due to the possibility of reducing the weight of the final part while maintaining mechanical properties comparable to components made entirely of metal. Conventional 3D printing processes do not enable the direct fabrication of hybrid structures consisting of solid metal and polymer parts due to the significant differences in the processing temperatures of both materials. A solution to this problem is the integration of two processes, electrodeposition and photopolymerization, which allow fabrication to be carried out at room temperature. This paper presents preparatory studies aimed at developing a new 3D printing technology that uses the simultaneous application of electrodeposition and photopolymerization to manufacture hybrid metal–polymer elements in a single, integrated 3D printing process. Here, a hybrid metal–polymer element is defined as a component composed of at least two bonded parts, including at least one metal part fabricated by electrodeposition and at least one polymer part produced by photopolymerization. Thus, it is not a polymer component merely coated with an electrodeposited metal layer, but a true hybrid structure consisting of functional metallic and polymeric parts. Such components can be manufactured using the world’s first hybrid 3D printer, which integrates electrodeposition and photopolymerization to produce metal–polymer hybrid parts within a single 3D printing process (the device has been submitted to the Polish Patent Office). However, its design and operating principle are beyond the scope of this paper. The presented research focuses on initial study of selected feedstock materials for this printer, namely photocurable resins and electroplating baths. Since the entire hybrid printing process occurs in an electroplating bath environment, studies of these materials for 3D printing under such conditions were essential. This work includes a screening study of photocurable formulations with respect to rheological properties, 3D printing tests in a model copper electroplating bath, and selection of a suitable bath brightener to maximize the quality (fine grain size, homogeneous grain distribution) of additively deposited copper layers. The study was conducted using copper electrodeposition and acrylate resin photopolymerization as model processes for evaluating the proposed hybrid metal–polymer 3D printing technology. Finally, the most suitable feedstock materials for producing metal–polymer hybrid parts via the proposed 3D printing method were selected.

## 1. Introduction

The hybrid metal–polymer components are advanced engineering structures that combine metal and polymer elements in a single, functionally integrated form. Their purpose is to merge the best properties of both material groups—strength, stiffness and conductivity of metals with the lightness, flexibility and vibration damping typical of polymers [1]. One of the dominant application areas for hybrid components is lightweight structures subjected to dynamic loads, primarily in the transportation sector. Other applications include protective and barrier elements in functional constructions, where environmental resistance is required while maintaining low weight [2]. Additionally, due to their ability to isolate vibrations and noise, they are used in HVAC (Heating, Ventilation and Air Conditioning) systems and devices sensitive to mechanical disturbances. In consumer electronics and mechatronic systems, hybrid components serve as housings, electromagnetic shields and frames that combine conductive and insulating properties [3,4]. Unlike classical composites reinforced with particles or fibers, these hybrids often appear as layered or spatially interconnected structures. Layered techniques, in which the joint is formed within the interface zone, include adhesive bonding, laser joining, ultrasonic welding and mechanical surface joining using conventional fasteners [3,4,5]. Spatially connected techniques, in which volumetric anchoring or a three-dimensional joint zone is created, include friction-based processes (such as friction spot joining, friction lap joining, friction riveting and friction-based filling), advanced mechanical anchoring methods (such as self-piercing riveting and clinching adapted to metal–polymer systems) and methods based on penetration of molten polymer into micro- and nanostructures of the metal surface [5].

3D printing, also known as additive manufacturing, is a process of creating physical objects from digital geometric models by successively depositing layers of material. Fabrication is performed directly from CAD (Computer-Aided Design) data, allowing precise replication of even the most complex three-dimensional structures. Owing to the layer-by-layer approach, it is possible to build geometries that would be difficult or even impossible to obtain using conventional manufacturing techniques [6,7]. Today, 3D printing enables the use of a wide range of materials, which contributes to its versatility and adoption across many industrial sectors [8]. The most commonly used materials include polymers, composites, metals, ceramics, smart materials (such as shape-memory alloys and polymers), as well as special materials (chocolate, meat, pasta). Plastics are most frequently used in 3D printing due to their availability and relatively simple processing compared to other engineering materials [7,8]. In polymer-based 3D printing, various forms of feedstock materials are employed, including thermoplastic filaments (e.g., PLA, ABS), polymer or metal powders and liquid photocurable resins [6,7,9,10,11]. Of particular relevance to the research presented in this paper are photocurable resins, which undergo rapid curing under UV/Vis irradiation, enabling high resolution, smooth surface finish and precise reproduction of complex geometries [12,13,14,15]. Their properties can be widely tailored through the selection of monomers, photoinitiators and additives (e.g., fillers or rheological modifiers), allowing adjustment of mechanical, optical and functional characteristics to meet specific application requirements [15,16,17,18].

The wide variety of polymer processing methods has led to the development of numerous 3D printing techniques [6,7,8,9]. The most widespread is Fused Deposition Modeling (FDM), classified as an extrusion-based method, in which thermoplastic material in the form of a filament is selectively deposited after being heated above its melting point and extruded through a moving printhead onto a heated build platform [19,20]. Another extrusion-based technique is Direct Ink Writing (DIW), which differs from FDM by not requiring the base material to reach its melting point [21]. VAT photopolymerization, including stereolithography (SLA) and Digital Light Processing (DLP), is one of the oldest and most precise 3D printing technologies [9,22,23]. It is classified as a photochemical method in which liquid photopolymer resins are selectively cured using a light source—typically ultraviolet (UV)—to build a three-dimensional object layer by layer [24]. Selective Laser Sintering (SLS) is used to manufacture parts from polymers and polymer composites by selectively sintering powder using a high-energy heat source, usually a CO_2_ laser [25]. Binder Jetting (BJ), unlike techniques such as SLS or FDM, does not use high temperature for particle bonding; instead, it relies on chemical or physical binding of powder particles by a liquid medium [26]. Metal-based 3D printing is less common than polymer-based printing due to high equipment costs, long production times, limited workspace and other factors. Most metal additive manufacturing techniques—Selective Laser Melting (SLM), Electron Beam Melting (EBM), Laser Metal Deposition (LMD) and Wire Arc Additive Manufacturing (WAAM)—use high-energy sources to melt metal [27,28,29]. Among low-temperature metal 3D printing processes, Binder Jetting is notable, though it does not permit the fabrication of solid metal parts [26], as well as technologies using electrochemical processes to produce solid metallic prints [30].

This paper presents screening research on a new 3D printing technology enabling the fabrication of hybrid metal–polymer components within a single integrated additive process. Such components can be manufactured using the world’s first hybrid 3D printer, which integrates electrodeposition and photopolymerization to produce metal–polymer hybrid parts (the device has been submitted to the Polish Patent Office). However, its design and operating principle are beyond the scope of this paper. Photopolymerization involves light-initiated polymerization of liquid resin, resulting in its localized curing and the formation of a layered polymer structure. Its advantages include high speed, point-specific action and the absence of a need for elevated temperature for polymerization to occur [6,7,8,13,14,16,17,31,32,33]. Electrodeposition, in turn, uses electrochemical phenomena to deposit metal on the surface of a cathode through current flow between the cathode and anode [30,34]. Combining both processes within a single technological platform enables direct formation of hybrid metal–polymer structures, in which metal elements are selectively built next to previously cured polymer segments without the need for additional assembly, machining or alignment steps. In the present manuscript, the focus is placed on investigating the two key processes, electrodeposition and photopolymerization, separately, but within the same electrolyte environment and under conditions representative of those occurring in the developed hybrid 3D printing system.

It should be clearly emphasized that, in the present paper, hybrid metal–polymer components are defined as parts consisting of at least one metallic component and at least one polymeric component, where both components are fabricated according to their predefined geometries (separately specified for each part) in successive stages of a single 3D printing process. Thus, these are not conventional composite materials produced by additive manufacturing, but rather structures composed of distinct parts that are manufactured separately and subsequently joined within a multi-material 3D printing process. The fabrication of such hybrid metal–polymer components has not been previously reported, primarily because existing metal 3D printing techniques rely on high-temperature processes, which would lead to the degradation or destruction of the polymer component.

Conventional techniques for manufacturing hybrid components, including layer-based methods (adhesive bonding, welding, mechanical joining) as well as approaches based on spatial anchoring, rely on joining previously fabricated metal and polymer elements through adhesive, mechanical or thermal interactions at the interface. As a result, the final joint properties are strongly dependent on surface quality, process parameters and joint geometry. Additionally, the design freedom of hybrid components produced using these methods is significantly limited [1,2,3,4]. In contrast, multi-material 3D printing that combines photopolymerization and metal electrodeposition enables the direct fabrication of integrated metal–polymer structures within a single process, eliminating the need for additional assembly steps characteristic of conventional joining techniques. Unlike adhesive bonding, welding or mechanical fastening, this approach results in a continuous and designable hybrid structure, which—through proper design—reduces stress concentrations and improves mechanical durability and reproducibility. Moreover, the process operates at low temperatures, thereby avoiding polymer degradation and issues related to thermal expansion mismatch that are typical for laser- or friction-based methods. Furthermore, this technology offers significantly greater freedom in geometric design and local distribution of metallic phases, which is difficult to achieve using conventional techniques. As a result, it enables the fabrication of functionally integrated structures with improved performance, higher precision and reduced weight compared to traditional hybrid manufacturing methods. The main limitation of this technology is the relatively low deposition rate of metals. High printing speeds (up to 0.5 kg/h for Cu) require strongly cathodic, modulated signals, which deteriorate surface quality, whereas milder potentials improve quality at the expense of deposition rate. Potential applications of the proposed hybrid metal–polymer 3D printing technique include lightweight structural–functional components with integrated electrical pathways, embedded electronics and sensors for aerospace systems and customized connectors combining mechanical and conductive functionalities. Additionally, the technology enables advanced energy devices, smart structures with integrated sensing and thermal management components through precise spatial control of metal–polymer architectures within a single manufacturing process.

On the other hand, the additive manufacturing of metal–polymer composite materials has emerged as a promising approach for fabricating multifunctional structures that combine the mechanical strength and electrical conductivity of metals with the lightweight, damping and processing versatility of polymers [35,36]. Among the most established techniques, fused deposition modeling (FDM) enables the fabrication of polymer matrices filled with metal particles, allowing the production of conductive or structurally reinforced components, although typically limited by relatively low metal content and anisotropic properties [37]. Powder bed fusion methods, such as selective laser sintering (SLS) and selective laser melting (SLM), have been adapted to process polymer–metal mixtures or to create polymer templates subsequently infiltrated or coated with metals, enabling higher structural complexity and improved mechanical performance [25,38].

Another important group of techniques includes direct ink writing (DIW), where viscoelastic inks containing metal particles or precursors are deposited layer-by-layer and subsequently cured or sintered, offering precise control over composition and architecture at multiple length scales [39]. VAT photopolymerization methods, such as stereolithography (SLA) and digital light processing (DLP), allow the fabrication of highly resolved polymer structures that can be metallized via electroless plating or electrodeposition, enabling the formation of conductive pathways within complex geometries [40,41]. Hybrid manufacturing approaches integrating photopolymerization with in situ electrodeposition have recently gained attention as a route toward simultaneous formation of polymer and metal phases within a single process environment [42].

In addition, directed energy deposition (DED) and cold spray techniques have been explored for embedding metallic features into polymer substrates, particularly for functional coatings and repair applications [43]. Despite these advances, key challenges remain related to interfacial adhesion, differences in thermal expansion and achieving homogeneous metal distribution within the polymer matrix [36,38]. Therefore, ongoing research focuses on process integration, advanced material formulations and multi-physics screening studies to enable reliable fabrication of high-performance metal–polymer hybrid structures for applications in electronics, aerospace and energy systems [35,42].

## 2. Experimental Section

### 2.1. Materials

The photocurable compositions selected for use in the new 3D printing technology were commercially available photopolymer resins intended for VAT photopolymerization. The first was Anycubic ABS-Like Resin V2, and the second was Anycubic Tough Resin 2.0. The selected resins exhibited low viscosity, which would make them unsuitable for 3D printing in an aqueous environment due to the loss of quality of the extruded material. Therefore, nanosilica thickening agent (silicon dioxide, spherical, porous nanopowder, 5–20 nm particle size, Sigma-Aldrich, St. Louis, MO, USA) was added to the resin. For rheological testing and 3D printing experiments in aqueous electrolyte solutions, samples of both resins were prepared containing 3 wt.%, 5 wt.% and 7 wt.% of nanosilica thickening agent, respectively. This was achieved by weighing 30 g, 50 g and 70 g of the thickening agent, as well as 970 g, 950 g and 930 g of the respective resin, followed by thorough mixing of the components using a mechanical stirrer.

**Figure 1:**
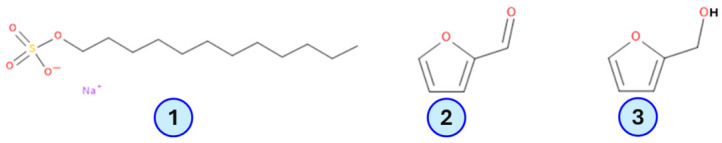
Structures of the brightening agents used. (1—SDS, 2—furfural, 3—furfuryl alcohol).

For preparing the electroplating baths, the following materials were used: copper(II) sulfate pentahydrate (Sigma-Aldrich), sulfuric acid (98%, Warchem, Zakręt, Poland) or sodium sulfate (Warchem) and brightening agents such as sodium dodecyl sulfate (SDS, Sigma-Aldrich), furfural (Sigma-Aldrich) and furfuryl alcohol (Sigma-Aldrich). The structures of the brightening agents are presented in Figure 1.

### 2.2. Apparatus

Rheological measurements were performed using a Physica MCR 302 rheometer (Anton Paar, Graz, Austria).

**Figure 2:**
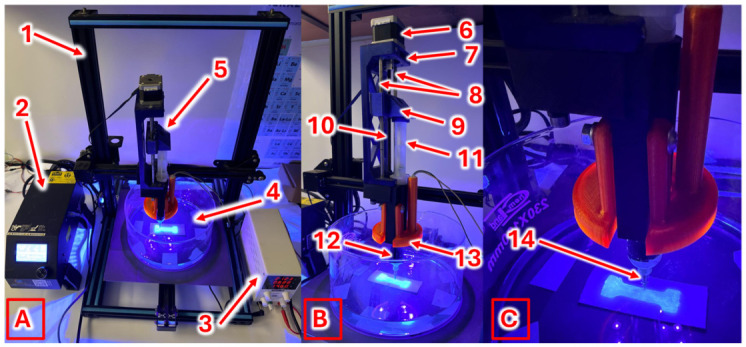
Photographs of the custom-built DIW 3D printer for printing in an aqueous environment (**A**), the extrusion system (**B**) and the printed sample (**C**) during the printing process (1—printer frame, 2—printer power supply, 3—UV-LED panel power supply, 4—electroplating bath container, 5—DIW printhead, 6—stepper motor, 7—DIW printhead frame, 8—linear guides, 9—carriage, 10—lead screw, 11—syringe plunger, 12—syringe, 13—UV-LED panel, 14—nozzle).

A custom-built DIW 3D printer dedicated for printing in an aqueous environment was constructed on the basis of a standard FDM-type 3D printer frame (Creality CR-10S, Shenzhen Creality 3D Technology Co., Ltd., Shenzhen, China) and equipped with a DIW printhead featuring a syringe from which the photocurable composite was extruded and subsequently cured using UV light delivered by a custom-built 405 nm UV-LED diode panel (AMPUL SYSTEM s.r.o., Šumperk, Czech Republic, Figure 2). The custom modification also involved using a flat-bottom container filled with an electroplating bath solution as the printing environment.

To determine the mass of the printed test samples, a standard analytical balance, the RADWAG AS 220.R2 PLUS, was used. The dimensions of these test samples were measured using a standard caliper manufactured by MITUTOYO (Kawasaki, Japan).

The prepared electroplating baths were subjected to electrochemical studies using the linear voltammetry technique with a PalmSens4 potentiostat. A glassy carbon electrode MF-2012 3.0 mm diameter (BASi, Lafayette, IN, USA) was used as the working electrode. The auxiliary electrode was a platinum spiral electrode MW-1033 (BASi), while the reference electrode was a silver–silver chloride electrode MF-2058 (BASi).

**Figure 3:**
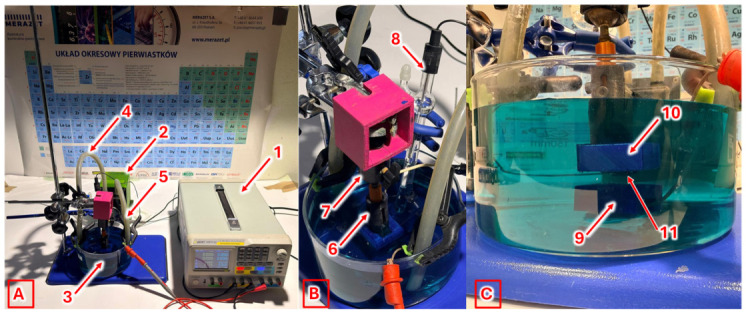
Experimental setup for copper electrodeposition in flow mode simulating the conditions of copper 3D printing by electrodeposition (**A**), the working area (**B**) and the electrode arrangement (**C**) in the electroplating bath (1—programmable DC power supply with potentiostat, 2—peristaltic pump, 3—electroplating bath container, 4—inlet hose, 5—outlet hose, 6—cathode mounting head, 7—ball joint, 8—Ag/AgCl reference electrode, 9—Pt anode, 10—Cu cathode, 11—electrodeposited copper layer).

The electrodeposition process was carried out using a dedicated experimental setup consisting of two mutually parallel electrodes: a copper cathode with a surface area of 1 cm^2^ and a platinum anode with a surface area of 1.5 cm^2^ (Figure 3). The electrodes were connected to a DC power supply equipped with a potentiostat and immersed in the electroplating bath solution contained in a reservoir. A programmable laboratory power supply—ODP3122 Owon 30 V 12 A (OWON, Zhangzhou, China)—was used, additionally equipped with a custom-built potentiostat designed for future application in a developed 3D metal printing device based on the metal electrodeposition process. Two hose connectors were mounted on the opposite side walls of the reservoir and connected with silicone tubing to a pump providing continuous electrolyte solution circulation (approximately 1 L/h). The connectors were positioned at a height selected to ensure that electrolyte solution flow occurred directly in the space between the electrodes. The distance between the electrodes was 1 cm. The use of this experimental setup made it possible to preliminarily reproduce the electrodeposition process conditions occurring during metal 3D printing. This was necessary to ensure that the results presented in this paper, concerning the composition of feedstock materials for electrodeposition-based 3D printing, can be applied to optimize the actual 3D printing process, which represents the key innovation of this study.

Microscopic cross-sectional and surface topography analysis of the electrodeposited metal was performed using a field-emission scanning electron microscope (FE-SEM/EDS, Hitachi S-4700 with a Noran System 7, Tokyo, Japan).

Photographs of the electrodeposited copper samples were taken using a DSX1000 optical microscope manufactured by OLYMPUS (Tokyo, Japan). The installed objective lens enabled 10× magnification of the sample area.

A universal testing machine—MTS Insight 50 (MTS Insight^TM^, Eden Prairie, MN, USA), equipped with a ±50 kN load cell and TestWorks 4.0 software—was used for the mechanical tests.

### 2.3. Methodology

#### 2.3.1. Rheology

The rheological properties of the photocurable compositions were examined at 25 °C using a 25 mm diameter spindle and a zero-gap setting of 0.1 mm. The measurement procedure began by placing a sample on the rheometer base and setting the zero gap. After achieving the required distance between the spindle and the substrate, excess material was removed, and the measurement was started. The tests were carried out at variable spindle rotational speeds (shear rates) ranging from 1/s to 100/s over a period of 550 s.

#### 2.3.2. DIW 3D Printing in Electroplating Bath Environment

First, the syringe mounted in the extruder was loaded with the prepared photocurable composite. Subsequently, the flat-bottom container was filled with a sulfate-based electroplating bath solution. Finally, the 3D printing process was initiated in a standard manner, analogous to conventional FDM-type printers. The printing process was performed by extruding the photocurable composite from a syringe mounted on a printhead moving in the horizontal plane (as in FDM-type printers) and curing it with UV light on the flat bottom of a container filled with the electroplating bath. The thickness of the electroplating bath layer above the printed test samples was approximately 1 cm. Samples fabricated using the DIW technique were in the form of test beams.

#### 2.3.3. Linear Voltammetry

The measuring vessel was filled with the solution of the investigated electroplating bath. The vessel was then placed in the measurement setup, and the electrodes were inserted into the electrolyte solution so that all electrodes were immersed at the same depth. Next, the system stirring and argon flow were initiated for 5 min. After this time, the stirring and argon flow were stopped, and the measurement was started. For the bath containing SDS, the potential range from 0 to −1.5 V was examined, whereas for the remaining baths the range from 0 to −1.1 V was investigated (with respect to the Ag/AgCl reference electrode). In each case, a potential scan rate of 100 mV/s was applied. After each measurement, the electrode was polished using diamond suspensions with particle sizes of 3 µm and 1 µm. Voltammetric tests were carried out for electroplating baths prepared as saturated copper sulfate solutions with a 1 M pH regulator/supporting electrolyte and different concentrations of brightener. The presented voltammetric measurements were intended for qualitative comparison of electrolyte systems rather than detailed kinetic analysis.

#### 2.3.4. Electrodeposition of Copper Layers

Copper plates with a surface area of 1 cm^2^ were subjected to electrodeposition in prepared sulfuric acid solutions containing pH regulator or supporting electrolyte and brightening agents for 20 min at a working potential of 2 V (for this measurement setup), corresponding to 0.3 V vs. Ag/AgCl. (according to the previously performed system calibration). Electrodeposition was performed under potentiostatic (constant potential) control. The measured current varied within the range of 0.4–0.6 A, depending on the stage of the process, which corresponds to an estimated current density of approximately 0.4–0.6 A/cm^2^, assuming a cathode surface area of 1 cm^2^. As illustrated in Figure 3, the process was conducted in a three-electrode configuration (8—Ag/AgCl reference electrode, 9—Pt anode, 10—Cu cathode) under flow conditions, with an electrolyte flow rate of 4 L/min. Samples fabricated by electrodeposition were therefore in the form of copper layers deposited on a square cathode surface (1 cm × 1 cm) with varying thickness, as shown in the Supplementary Materials. In preliminary tests (outside the scope of this paper), the bubble accumulation effect was observed. However, in the experiments described in this study, the application of electrolyte flow at a relatively high rate (4 L/min) completely eliminated this effect. This occurred already at a flow rate corresponding to approximately one quarter of the value ultimately selected as optimal. Under flow conditions, gas bubbles were effectively removed almost immediately by the electrolyte stream, preventing their accumulation on electrode surfaces.

Afterwards, the samples were rinsed with distilled water and methyl alcohol. The composition of the prepared solutions (electroplating baths) is presented in Table 1.

#### 2.3.5. SEM/EDS

SEM images were obtained at an accelerating voltage of 20.0 kV and a working distance of approximately 13.2–13.3 mm. The images were recorded at different magnifications ranging from approximately ×30 to ×350, which allowed observation of both the overall morphology of the layer and the details of the surface microstructure. Both the surface of the samples and their cross-sections were analyzed, and the image scale bars ranged from 100 µm to 1 mm.

#### 2.3.6. Tensile Behavior

Tensile tests of samples 3D-printed using Anycubic Tough Resin 2.0 in saturated copper sulfate solution were performed in accordance with ISO 527 [44], using mechanical clamping grips and anti-slip inserts to prevent specimen slippage. The crosshead speed was 10 mm/min, and the initial gauge length was set to 35 mm. All tests were carried out under controlled laboratory conditions (22 ± 1 °C, relative humidity ~40%), and the specimens were conditioned for at least 24 h prior to testing. For each material variant, three specimens were tested to ensure reproducibility of the results. The Young’s modulus and tensile strength were determined from the recorded stress–strain curves. The reported values represent the mean ± standard deviation. Since the behavior of the Anycubic ABS-like and Tough resins was similar, the Anycubic Tough resin was selected to investigate the effect of the addition of a thickening agent and the applied 3D printing environment in the form of an electroplating bath on the mechanical properties of the printed samples, taking into account its good mechanical strength as declared by the manufacturer.

## 3. Results

### 3.1. Rheological Studies

**Figure 4:**
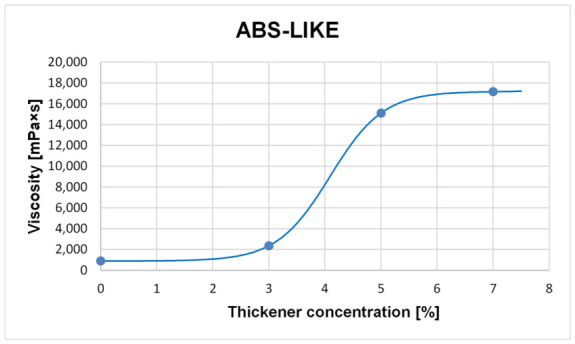
Chart showing the dependence of the viscosity of an ABS-Like resin-based composition on the concentration of the nanosilica thickening agent.

**Figure 5:**
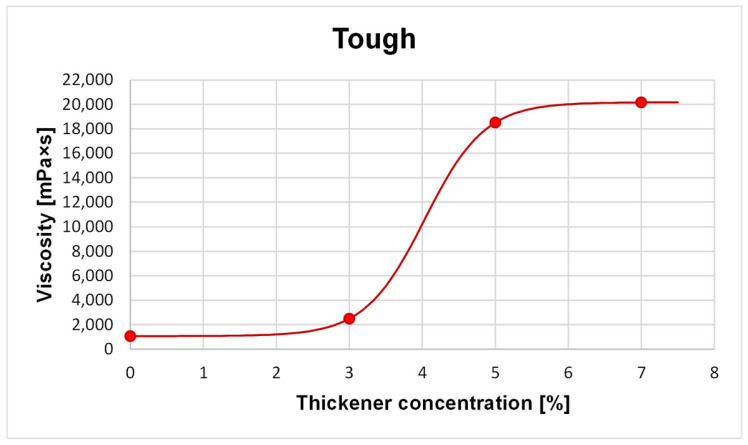
Chart showing the dependence of the viscosity of a Tough resin-based composition on the concentration of the nanosilica thickening agent.

The first investigation involved examining the rheological properties of the photocurable compositions intended for 3D printing. For this purpose, eight formulations were prepared using ABS-Like Resin V2 and Tough Resin 2.0, each containing 0%, 3%, 5% or 7% silica-based thickener by mass. These studies made it possible to determine the effect of the thickener content on the viscosity of the prepared photocurable compositions (Figure 4 and Figure 5).

Figure 4 and Figure 5 show the dependence of the composition viscosity on the thickener content (wt.%), for the ABS-like and Tough compositions, respectively. For both photocurable resins, a significant increase in viscosity is observed for compositions containing 5 wt.% thickener compared to samples with 3 wt.%. It is worth noting that samples containing more than 5 wt.% behave like solids under low shear stress conditions, which suggests the presence of a yield point or a stiffened internal structure. It should be emphasized that the observed liquid-to-solid transition corresponds to a rheological transition associated with the formation of a percolated particle network rather than a true thermodynamic phase transition. Therefore, the apparent discrepancy between qualitative observations and the plotted viscosity trend results from the inherently continuous character of the measurement.

### 3.2. Screening Study of Process Parameters for 3D Printing Using Photocurable Resins in Aqueous Electrolyte Solutions

In the next stage of the study, the process parameters for 3D printing with photocurable compositions were optimized. The first step was selecting an appropriate nozzle diameter in the range of 0.2 mm to 2.0 mm. Preliminary tests showed that the best results in terms of print quality and extrusion efficiency were achieved using nozzles with diameters of 0.4 mm and 0.5 mm. Both diameters enabled smooth and stable extrusion of the prepared photocurable compositions while maintaining high print resolution.

**Figure 6:**
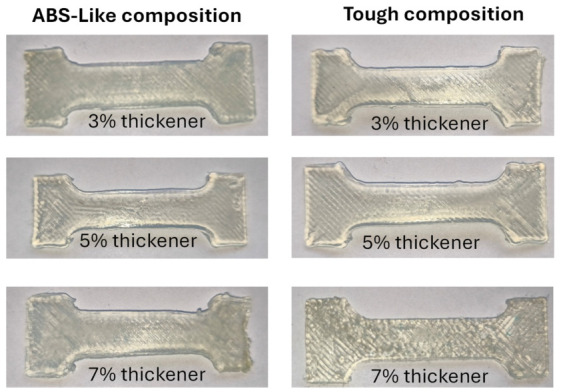
Test samples 3D-printed in a saturated copper sulfate solution acidified with 1 mol of sulfuric acid, using the investigated photocurable compositions with different thickener concentrations.

Simultaneously, the “slicer” software settings were adjusted to the newly installed extruder. Particular attention was given to selecting an appropriate printhead travel speed, as excessive movement could disrupt the surface of the electrolyte solution, potentially causing the solution to spill onto the 3D printer. Tests demonstrated that the maximum printhead speed that ensured liquid stability was 20 mm/s. This value was adopted as the standard for subsequent experiments. Next, the optimal extrusion multiplier, equal to 1.20, was established, controlling the rotational speed of the stepper motor dispensing the photocurable composition. After completing device calibration and verifying all settings, the main experimental stage commenced: performing 3D printing of test samples using prepared photocurable formulations directly in the electroplating bath environment. The electroplating bath consisted of a saturated copper sulfate solution with the addition of one mole of sulfuric acid. Figure 6 presents photographs of the test samples 3D-printed using the analyzed compositions in an electroplating bath environment.

The masses and dimensions of the 3D-printed test samples are summarized in Table 2. In all cases, the measured values are lower than the theoretical ones, indicating systematic underfilling during printing in the electrolyte environment.

The systematically lower measured mass compared to the theoretical value can be attributed to two main factors related to the printing process conducted in a liquid electrolyte environment:

1—Local discontinuities in infill resulting from the interaction between resin extrusion and the surrounding liquid medium. These may have arisen from the detachment of fragments of the extruded resin track (prior to full curing) and their subsequent transport into the bulk of the electroplating bath.

2—Under-extrusion effects. The selected extrusion multiplier may not have been optimal in all cases, and due to flow resistance during resin extrusion from the reservoir, the actual amount of extruded material may have been slightly lower than expected.

For both material systems, the thickener content plays a key role in determining geometric accuracy and structural integrity. In the ABS-Like series, the 5 wt.% formulation yields the most uniform morphology and highest dimensional stability, whereas 3 wt.% and 7 wt.% lead to increased irregularities due to insufficient structural support and excessive viscosity, respectively. A comparable tendency is observed for the Tough resin, where the 5 wt.% composition provides the best dimensional fidelity and most homogeneous structure. In contrast, the 3 wt.% samples exhibit visible track formation and reduced stability, while the 7 wt.% samples show pronounced roughness and discontinuities, consistent with over-thickening effects.

Overall, an intermediate thickener concentration (≈5 wt.%) offers the most favorable balance between flowability and shape retention, resulting in the highest print quality irrespective of the resin type.

The discrepancies between measured and theoretical values can be attributed primarily to polymerization shrinkage. The largest relative deviations are observed for the thickness (h), reaching approximately 8–21%, indicating high sensitivity to processing conditions and the influence of the aqueous environment, which promotes slight flattening of the structures. In contrast, in-plane dimensions (a, b_1_, b_2_) remain more accurate, typically within 2–5%. The smallest mass deviations are noted for the Tough 3% and 5% samples.

The best overall performance—considering dimensional accuracy, layer continuity and structural uniformity—is consistently achieved for compositions containing 5 wt.% thickener. These samples exhibit stable layer deposition without visible defects, reflecting an optimal interplay between rheological properties and processing parameters. No significant differences are observed between the two resin systems, suggesting that thickener concentration is the dominant factor governing print quality. The poorest results occur at 7 wt.%, where excessive viscosity limits material extrusion and interlayer bonding, leading to structural defects.

A more detailed analysis of the defects observed in the 3D-printed samples reveals that they can be classified into several distinct categories, each associated with specific physical mechanisms occurring during printing in the electrolyte solution environment.

First, the non-uniform layered structure, visible as irregular deposition tracks and local variations in layer thickness, is primarily related to insufficient stabilization of the extruded resin track in the liquid medium. At low thickener content (3 wt.%), the viscosity of the photocurable composition is too low to maintain the shape of the deposited resin track, leading to lateral spreading and partial merging of adjacent tracks. This results in a heterogeneous internal structure and uneven material distribution. In contrast, at high thickener content (7 wt.%), excessive viscosity limits proper flow and wetting of the previously deposited layer, leading to incomplete interlayer bonding and the formation of local discontinuities.

Second, the occurrence of blurred or poorly defined edges is attributed to the combined effects of fluid–structure interaction and limited curing rate. During deposition, the surrounding electrolyte solution exerts shear forces on the extruded material, causing deformation of the resin track before full photopolymerization occurs. This effect is particularly pronounced at the outer contours, where the lack of neighboring material reduces geometric confinement. As a result, edge rounding and loss of dimensional sharpness are observed. This phenomenon is further enhanced by polymerization shrinkage, which contributes to edge recession and dimensional undershoot, especially in the vertical (h) direction.

Third, local discontinuities and surface irregularities, especially evident for samples containing 7 wt.% thickener, are associated with unstable extrusion conditions. High viscosity increases the resistance to flow through the nozzle, leading to pulsation of the extruded material and intermittent deposition. This results in the formation of gaps, surface roughness and locally weakened regions within the structure. Additionally, increased nanosilica content may lead to light scattering during UV exposure, reducing curing efficiency and contributing to microstructural heterogeneity.

A key observation is that the severity of all identified defect types strongly depends on the balance between viscous forces, surface tension effects and curing kinetics. The electrolyte solution environment introduces additional complexity compared to conventional DIW or FDM printing, as it modifies both the hydrodynamic conditions and heat/mass transfer processes at the interface.

The optimal behavior observed for compositions containing 5 wt.% thickener can be explained by achieving a critical viscosity range, in which the extruded resin track retains its shape while still allowing sufficient flow for proper interlayer adhesion. In this regime, the influence of external fluid forces is minimized, extrusion remains stable, and photopolymerization proceeds efficiently, leading to improved geometric accuracy and reduced defect density.

From a process screening study perspective, the presented analysis indicates that defect minimization in hybrid electrochemical 3D printing requires simultaneous control of (1) rheological properties of the photocurable composition, (2) extrusion parameters (flow stability) and (3) curing conditions (light intensity and exposure time). Further improvements may be achieved by tailoring the curing kinetics to match the characteristic timescale of resin track deformation in the electrolyte solution, as well as by optimizing flow conditions in the electroplating bath to reduce hydrodynamic disturbances.

### 3.3. Voltammetry

**Figure 7:**
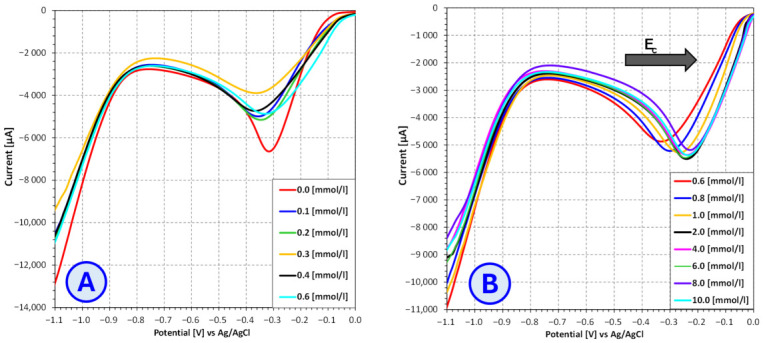
Influence of **SDS** concentration on the current–voltage characteristics of copper sulfate and sulfuric acid-based electroplating baths for **SDS** concentrations ranging from 0.0 mmol/L to 0.6 mmol/L (**A**) and from 0.6 mmol/L to 10.0 mmol/L (**B**).

**Figure 8:**
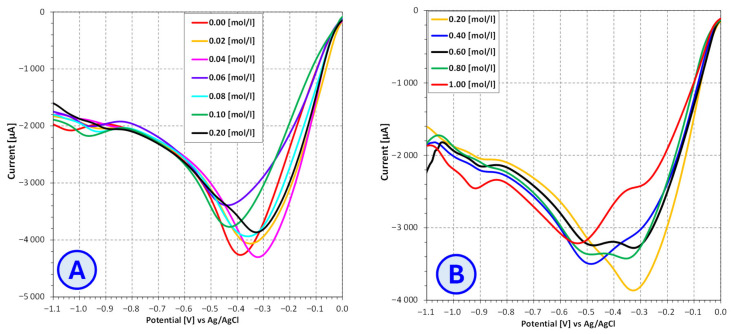
Influence of **furfural** concentration on the current–voltage characteristics of copper sulfate and sodium sulfate-based electroplating baths for **furfural** concentrations ranging from 0.00 mol/L to 0.20 mol/L (**A**) and from 0.20 mol/L to 1.00 mol/L (**B**).

**Figure 9:**
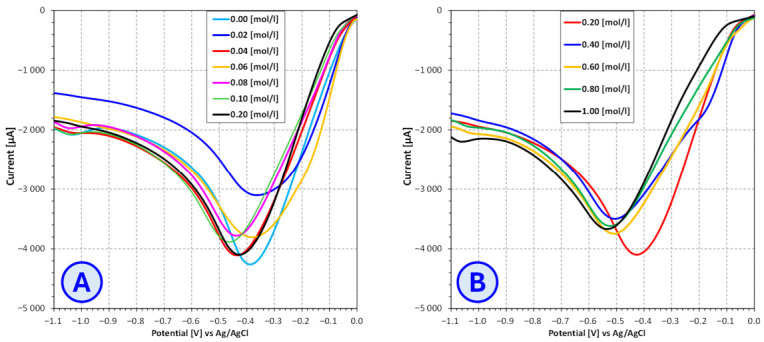
Influence of **furfuryl alcohol** concentration on the current–voltage characteristics of copper sulfate and sodium sulfate-based electroplating baths for **furfuryl alcohol** concentrations ranging from 0.00 mol/L to 0.20 mol/L (**A**) and from 0.20 mol/L to 1.00 mol/L (**B**).

In order to determine the optimal voltage for conducting the copper electrodeposition process from electroplating baths containing different brightener additives (SDS, furfural, furfuryl alcohol), the current–voltage characteristics of these baths were determined using the linear voltammetry technique (Figure 7, Figure 8 and Figure 9).

The results of the study on the influence of sodium dodecyl sulfate (SDS) on the copper-ion reduction potential revealed a concentration-dependent effect of this compound within the electroplating bath. In the low-concentration range, from 0.0 mmol/dm^3^ to 0.6 mmol/dm^3^, no noticeable shifts were observed in the signal corresponding to the reduction process. The only detectable change was a slight decrease in signal intensity, which may indicate a partial weakening of the electrochemical process, although without affecting its reduction potential. This effect may be associated with the initial adsorption of SDS on the electrode surface, which at this stage is insufficiently strong to modify the reduction potential.

As the SDS concentration increased to the range of 0.6–10.0 mmol/dm^3^, a gradual shift in the copper-ion reduction signal toward more positive potentials was observed. This phenomenon may indicate increasingly strong interactions between the surfactant and the electrode surface, leading to alterations in the local electrochemical environment. The presence of SDS may influence ion transport to the cathode surface and promote the formation of an ordered adsorbed layer, thereby modifying the conditions under which the electrode reaction proceeds. As a surfactant, SDS may facilitate the transport of Cu^2+^ ions toward the electrode by lowering surface tension and potentially altering ion mobility, which could increase the availability of Cu^2+^ for the reaction, ultimately contributing to the shift in the reduction potential toward more positive values.

The analysis of the influence of furfural on the copper-ion reduction potential revealed no clear or systematic trend in the electrochemical parameters. Furfural did not exhibit a consistent direction of interaction; depending on its concentration and the measurement conditions, both shifts toward more positive and more negative reduction potentials were observed. This may indicate unstable behavior of this compound within the electrolyte solution environment, as well as the possible influence of factors such as electrode contact time, solubility or the nature of its interactions with metal ions.

Based on the observed changes in the copper-ion reduction potential and alterations in the voltammogram shape—including the appearance of a second reduction signal—it may be postulated that several overlapping effects are involved. These include changes in the solubility of bath components, complex formation or copper-ion reduction processes similar to those reported for thiourea. Furfural may also form weak complexes with Cu^2+^ through the oxygen atom of its carbonyl group. Furthermore, due to the presence of an aldehyde group, furfural exhibits reducing properties. In the presence of Cu^2+^ ions, it may undergo a redox reaction in which furfural is oxidized while Cu^2+^ ions are reduced to Cu^+^. Although this process proceeds slowly in an acidic environment, it nonetheless occurs. The color change observed in stored solutions confirms that this reaction takes place in the studied systems.

Notably, for furfural concentrations ranging from 0.4 mol/dm^3^ to 1.0 mol/dm^3^, a physical phenomenon was observed in which furfural separated from the electrolyte solution after standing. Due to its limited solubility in aqueous electrolyte solution, furfural formed a clearly visible layer on the surface of the solution, indicating that its solubility limit had been exceeded. Such behavior may significantly affect the reproducibility and interpretation of electrochemical results because of the variable availability of furfural molecules within the solution and the potential formation of phase layers at the electrode–solution interface.

The analysis of the influence of furfuryl alcohol on the reduction potential of copper ions revealed a concentration-dependent and heterogeneous behavior of this compound within the electrolyte solution. In the low-concentration range, from 0.00 mol/dm^3^ to 0.04 mol/dm^3^, no significant changes in the reduction potential were observed. This indicates either limited electrochemical activity of furfuryl alcohol at low concentrations or insufficient interaction with the electrode surface and Cu^2+^ ions in the studied system.

As the concentration increased to 0.04–0.20 mol/dm^3^, a distinct shift in the reduction potential toward more negative values was recorded. Within this concentration range, the potential stabilized at a lowered level, with only minor deviations. Further increases in concentration, from 0.20 to 1.00 mol/dm^3^, led to an additional shift in the reduction potential toward even more negative values, followed again by stabilization. This behavior suggests the emergence of another saturation threshold in which the effects associated with a high abundance of furfuryl alcohol dominate, potentially resulting in more persistent interactions with the electrode or modifications to the properties of the diffusion layer.

The results suggest that furfuryl alcohol affects the reduction potential of Cu^2+^ in a gradual, concentration-dependent manner. Two ranges of pronounced activity can be distinguished: the first between 0.04 and 0.20 mol/dm^3^ and the second between 0.20 and 1.00 mol/dm^3^, each characterized by stabilization of the reduction potential. It is worth noting that one of the possible phenomena occurring when furfuryl alcohol is used as a brightener is its oxidation to furfural (an aldehyde), followed by further oxidation of furfural, as described earlier. In these reactions, Cu^2+^ ions are reduced to Cu^+^, similarly to the mechanism observed when furfural is used as a brightener. As in the case of furfural, furfuryl alcohol may also form complexes with Cu^2+^ ions. The superposition of these effects, along with the influence of various parameters on their intensity, means that the precise impact of furfuryl alcohol on the redox characteristics of the electroplating bath depends on many factors and is difficult to predict.

To elucidate the mechanisms governing the influence of individual brighteners on the reduction potential of metal ions in electroplating baths, it is essential to examine the behavior of the brightener itself within the studied potential range and under the specific environmental conditions. Some brighteners may adsorb onto the electrode surface via classical surface adsorption, whereas in other cases, redox reactions of the brightener at the electrode or redox interactions between the brightener and the metal ions in solution may occur. The formation of metal–brightener complexes is also possible, which could further contribute to shifts in the reduction potential. For this reason, in the subsequent stage of work on the developed technology—beyond the scope of the present paper—such measurements will be performed using voltammetric techniques.

### 3.4. SEM Analysis of Electrodeposited Copper Layers

**Figure 10:**
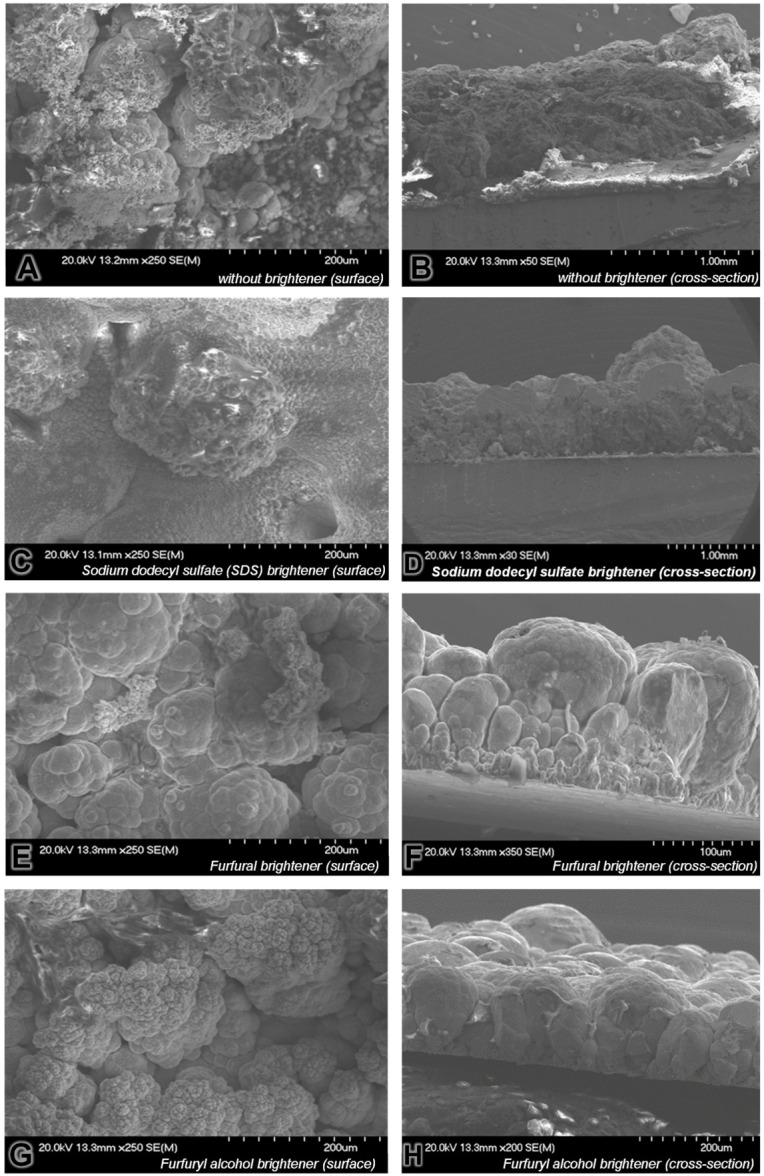
SEM images of the surfaces of electrodeposited copper layers obtained using electroplating baths without brightening agent (**A**,**B**) and with various brighteners (**C**–**H**) on the surface of the sample (**on the left side**) and in its cross-section (**on the right side**).

Copper sulfate electroplating baths without a brightener and with various brighteners were used to prepare the electrodeposited copper layers. SEM images of the surfaces and cross-sections of electrodeposited copper layers obtained from electroplating baths without a brightener and with various brighteners are presented in Figure 10.

The copper layer electrodeposited from the plating bath without the addition of a brightener is characterized by a highly developed and irregular surface morphology (Figure 10A). Large agglomerates with diameters of approximately 50–150 µm predominate, locally reaching 200–300 µm. The grain size distribution is very broad, indicating a lack of control over nucleation and crystal growth processes. The surface exhibits pronounced porosity and the presence of intergranular voids. Marked non-uniformity in the growth rate across different regions is observed, together with local dendritic and cauliflower-like structures. The coalescence of secondary nuclei on the surface of previously formed grains is clearly visible, confirming an unstable course of electrocrystallization. In terms of the cross-section, the copper layer deposited without a brightener (Figure 10B) reveals highly irregular thickness, a strongly developed and rough interfacial zone near the substrate and the presence of internal voids and microcracks within the deposit. No distinct, compact columnar structure is observed. The growth mechanism is predominantly island-like and dendritic, leading to the formation of closed pores and weak adhesion to the substrate. The structure is markedly loose and prone to delamination. Such morphology is typical of deposition under conditions of limited kinetic control over Cu^2+^ ion reduction.

The addition of SDS to the plating bath results in a partial improvement in surface homogeneity (Figure 10C). Grains with diameters of 10–40 µm are observed in certain regions, alongside larger agglomerates of 80–150 µm in others. A clear reduction in porosity and a more compact morphology are evident compared to the sample obtained from the bath without a brightener. As a surfactant, SDS improves substrate wettability and modifies surface tension, thereby promoting an increased number of nucleation sites. However, localized regions of accelerated growth are still present, indicating a non-uniform effect of the SDS additive. The cross-sectional image of the copper layer deposited from the SDS-containing bath (Figure 10D) shows increased compactness, a partially developed columnar structure and a reduced number of large voids. A more uniform layer thickness is also observed. The growth mode becomes more continuous, although locally porous regions persist. The structure is distinctly more compact than that of the copper layer deposited without additives, yet it is not fully fine-grained.

The copper layer deposited from the electrolyte solution containing furfural (Figure 10E) exhibits a strongly developed cauliflower-like morphology, dominated by grains of 40–120 µm and large agglomerates of 150–200 µm. The grain structure remains coarse and heterogeneous. Intensive secondary overgrowth on previously formed grains is visible, suggesting diffusion-controlled growth and local current density overshoot. The cross-section (Figure 10F) reveals pronounced columnar growth, the presence of dendritic features, significantly varied layer thickness and local microporosity. The structure is less compact than that obtained with SDS. A well-developed directional growth perpendicular to the substrate is evident, indicating limited inhibition of axial growth by furfural.

The copper layer electrodeposited from the bath containing furfuryl alcohol (Figure 10G) shows a surface dominated by grains of 30–100 µm, with a fraction of 150–200 µm grains. The structure is more compact than that obtained with furfural. Although some heterogeneity remains, it is clearly reduced. The morphology is significantly more compact than in the absence of a brightener or with furfural addition, though it still retains a coarse-grained character. In terms of the cross-section (Figure 10H), a relatively dense layer with fairly uniform thickness, a limited number of voids and a partially developed, more regular columnar structure can be observed. The growth process appears more controlled than in the case of furfural, suggesting a stronger influence of furfuryl alcohol on adsorption phenomena and the blocking of active growth sites.

In summary, the analysis of the morphology of the electrodeposited copper layers revealed clear differences in both grain size and distribution uniformity, directly resulting from the type of brightening agent used in the electrodeposition process. The best structural quality, defined by the highest compactness, greatest cross-sectional uniformity and lowest porosity, was achieved with the addition of furfuryl alcohol. It may be hypothesized that, due to the dense and relatively homogeneous microstructure of copper layers electrodeposited from the bath containing furfuryl alcohol, 3D-printed components fabricated using such an electrolyte solution would exhibit improved mechanical strength, reduced susceptibility to crack initiation, increased hardness and enhanced fatigue resistance compared to those obtained with the other analyzed brighteners. Nevertheless, the resulting structure remains too coarse-grained (30–100 µm, locally 150–200 µm) for the investigated furfuryl alcohol-containing plating bath to be directly applied as a final feedstock for copper electrochemical 3D printing. Components produced under these conditions would likely exhibit insufficient dimensional accuracy for practical applications and would not be competitive with commercial metal additive manufacturing techniques, such as those based on metallic powder sintering. Therefore, further research is required to identify more effective brighteners suitable for plating bath intended as feedstock materials for metal electrodeposition-based 3D printing technologies.

**Figure 11:**
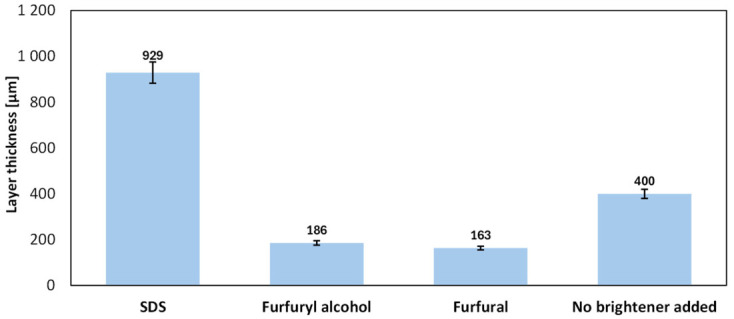
Bar chart of the thickness of electrodeposited copper layers for all analyzed samples (thickness determined based on cross-sectional SEM image analysis).

Based on the analysis of cross-sectional SEM micrographs, the thicknesses of the electrodeposited copper layers were determined for all investigated samples. The obtained values are summarized in Figure 11.

A strong dependence of the layer thickness on the applied bath additive was observed. The electrolyte solution containing SDS produced the thickest deposit (~929 μm), i.e., approximately 2.3 times thicker than the layer obtained without a brightener (~400 μm), indicating a substantial increase in the effective deposition rate and current efficiency. In contrast, the furan-based additives significantly reduced the deposit thickness (furfural: ~163 μm; furfuryl alcohol: ~186 μm), corresponding to a decrease of approximately 54–59% relative to the additive-free bath. This behavior is consistent with an inhibition mechanism involving the adsorption of organic compounds at active growth sites, resulting in suppressed layer growth, potentially accompanied by improved surface leveling and grain refinement [30,34]. It may be concluded that although the addition of furfuryl alcohol yields the best structural quality, it markedly slows down the electrodeposition process to such an extent that this plating bath cannot be practically applied for 3D printing. Considering both structural quality and deposition rate, the SDS-containing bath appears to be the most suitable among the analyzed plating bath for application in electrochemical 3D printing.

### 3.5. EDS Analysis of Electrodeposited Copper Layers

**Figure 12:**
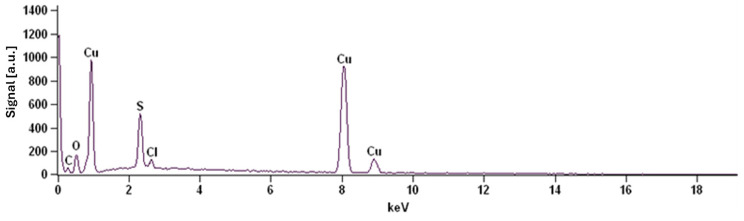
EDS spectrum of the sample deposited from the bath containing SDS brightener.

Figure 12 shows a representative EDS spectrum of the copper layer deposited from the plating bath containing SDS.

The elemental composition (mass fractions, wt.%) obtained from SEM/EDS analyses for each electrodeposited copper surface is presented in Table 3.

The EDS spectral analysis demonstrated that all obtained coatings consist predominantly of copper (Cu), whereas the differences between the samples mainly concern the oxygen (O) and sulfur (S) contents, with trace amounts of chlorine (Cl) and carbon (C). These differences primarily result from the type of brightener used and the composition of the plating bath.

The copper layer obtained from the bath containing SDS exhibited the lowest Cu content (~78.8 wt.%) and the highest O (~12 wt.%) and S (~7.9 wt.%) contents. This indicates strong adsorption of the sulfur-containing additive and/or the presence of residual sulfate species from the plating bath on the deposit surface. The elevated oxygen content further suggests a higher contribution of copper oxides and/or adsorbed electrolyte solution residues.

The copper layers deposited from baths containing furfuryl alcohol and furfural showed significantly higher Cu contents (~90.9 wt.% and ~93.3 wt.%, respectively) and lower O and S levels. In particular, the furfural-containing sample exhibited the highest metallic purity. Since these additives do not contain sulfur, the detected S most likely originates from sulfate anions present in the CuSO_4_-based plating bath. The lower O and S contents indicate a more compact and smoother deposit structure with a reduced amount of entrapped plating bath residues.

The copper layer deposited without a brightener contained a moderate Cu content (~83.5 wt.%) along with relatively high O (~8.1 wt.%) and S (~7.7 wt.%) contents. This may be associated with increased surface roughness and porosity, which facilitate electrolyte solution retention and accelerate surface oxidation.

The presence of oxygen in all analyzed samples is typical and can be attributed to thin copper oxide layers (Cu_2_O, CuO) formed upon exposure to air. Sulfur most likely originates from residual sulfate ions (SO_4_^2−^) in the electrolyte solution and from the adsorption of sulfate-containing additives (e.g., SDS). Trace amounts of chlorine may result from chemical impurities, rinsing water or sample preparation procedures.

In the case of the Cu-ETP reference sample, approximately 10 wt.% C was detected, which is most likely associated with a surface layer of organic contaminants.

In summary, the applied brightener significantly influences the surface chemical composition of the deposited copper. The highest chemical purity was achieved for the bath containing furfural, whereas the use of SDS resulted in the highest fraction of non-copper elements (O and S), most likely due to additive adsorption and plating bath residue retention.

From the perspective of the properties of potential 3D-printed components fabricated via additive manufacturing based on electrodeposition, the most advantageous plating bath in terms of chemical purity are those containing furfural or furfuryl alcohol. Elevated concentrations of non-copper elements, such as sulfur, as well as higher fractions of copper oxides, may adversely affect properties of copper prints, including their mechanical strength and electrical conductivity.

### 3.6. Tensile Behavior

**Figure 13:**
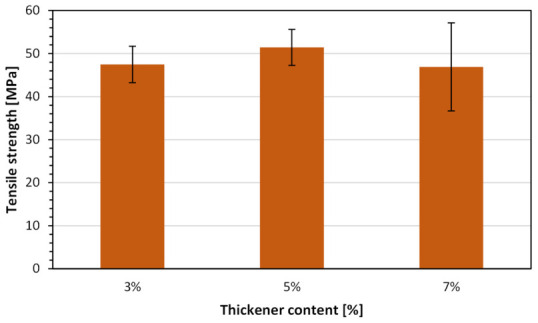
Comparison of the tensile strength of the analyzed test samples 3D-printed in a saturated copper sulfate solution acidified with 1 M sulfuric acid, using Anycubic Tough Resin 2.0 with different thickener concentrations.

**Figure 14:**
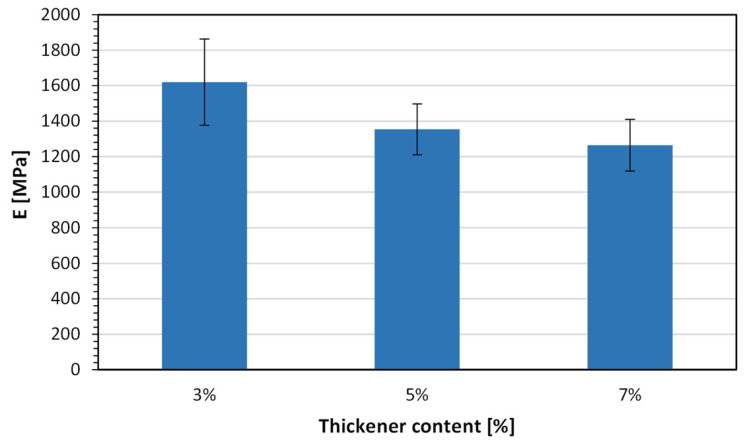
Comparison of the Young’s modulus values for the analyzed test samples 3D-printed in a saturated copper sulfate solution acidified with 1 M sulfuric acid, using Anycubic Tough Resin 2.0 with different thickener concentrations.

Figure 13 and Figure 14 show a comparison of the tensile strength and Young’s modulus values of the analyzed test samples 3D-printed in a saturated copper sulfate solution acidified with 1 M sulfuric acid, using the investigated photocurable compositions with different thickener concentrations.

A clear dependence of mechanical properties on the thickener content is observed. The tensile strength exhibits a non-monotonic trend, with the highest value recorded for the composition containing 5 wt.% of the thickener (≈51 MPa), compared to ≈47 MPa for 3 wt.% and ≈46–47 MPa for 7 wt.%. This indicates the existence of an optimal thickener concentration that enhances mechanical performance. The improvement at 5 wt.% can be attributed to increased viscosity and improved shape retention during printing in the liquid electrolyte solution environment, which likely reduces structural defects and promotes more uniform crosslinking. In contrast, further increasing the thickener content to 7 wt.% does not lead to additional strengthening and may even slightly reduce tensile strength, potentially due to hindered photopolymerization kinetics, increased light scattering or the formation of microstructural heterogeneities.

In the case of Young’s modulus, a decreasing trend with increasing thickener concentration is observed. The highest modulus is obtained for the 3 wt.% composition (≈1600 MPa), followed by 5 wt.% (≈1350 MPa) and 7 wt.% (≈1250 MPa). This suggests that increasing the thickener content leads to a reduction in stiffness, likely due to the introduction of a more heterogeneous or less densely crosslinked polymer network. The presence of higher amounts of nanosilica may also interfere with polymer chain mobility during curing, resulting in a structure that is less rigid despite maintaining comparable strength.

The observed trends indicate a trade-off between strength and stiffness as a function of thickener concentration. While moderate addition (5 wt.%) improves tensile strength, lower concentrations (3 wt.%) are more favorable for maximizing stiffness. Importantly, the relatively small standard deviations in both tensile strength and modulus values confirm good repeatability of the process, even under the challenging conditions of printing in a conductive electrolyte solution environment.

Overall, the results demonstrate that the incorporation of a nanosilica thickener is essential for enabling stable 3D printing in an electrochemical bath. However, its concentration must be carefully optimized. A content of approximately 5 wt.% appears to provide the best compromise between mechanical strength and processability, making it the most suitable formulation for further development of hybrid metal–polymer structures fabricated via combined photopolymerization and electrodeposition.

The tensile strength values obtained in this study (46–51 MPa) fall within the typical range reported for standard and tough photocurable resins used in SLA/DLP technologies (approximately 40–60 MPa), indicating comparable mechanical performance despite processing in an electrolyte solution environment [40,45]. In contrast, the Young’s modulus values (1.25–1.6 GPa) are slightly lower than those commonly reported for rigid photopolymer resins (typically 2.0–2.5 GPa), suggesting a reduction in stiffness likely associated with the presence of the thickening agent and the specific photopolymerization conditions in the electrochemical bath [35,40,45].

## 4. Conclusions

Based on the conducted preliminary studies, key material and process parameters necessary for the development of additive manufacturing technology for hybrid metal–polymer components using coupled photopolymerization and metal electrodeposition processes were identified. The obtained results constitute the screening study of feedstock materials intended for the developed 3D printing technology.

The results demonstrated that DIW 3D printing of photocurable resins in electroplating baths under flow conditions can be successfully performed using commercially available DLP printing resins, provided that their rheological properties are appropriately modified. The addition of a thickening agent significantly increased the viscosity of the resin compositions. An optimal balance between extrudability and shape stability was achieved for compositions containing approximately 5 wt.% thickener, enabling printing quality comparable to conventional DIW or FDM processes.

Experiments conducted directly in the electrolyte solution confirmed the feasibility of polymer printing in an electroplating bath, which is a key prerequisite for the implementation of integrated manufacturing of hybrid metal–polymer structures. Dimensional analysis of the printed samples revealed deviations from theoretical values mainly due to polymerization shrinkage and the influence of the aqueous environment, with the largest deviations observed in the axial direction, while the dimensional accuracy in the XY plane remained relatively good.

Electrochemical studies using linear sweep voltammetry showed that the applied brighteners significantly influence the Cu^2+^ reduction process in the analyzed plating bath. The addition of SDS shifted the reduction potential toward more positive values, while furfuryl alcohol caused a shift toward more negative potentials. In the case of furfural, the relationship between concentration and reduction potential was less pronounced, suggesting a more complex interaction mechanism.

Morphological analysis of the electrodeposited copper layers confirmed a strong influence of the brightener type on the microstructure of the deposits. The most porous structures were obtained without additives, whereas the use of SDS improved the structural uniformity and increased the deposition rate. The most compact morphology was observed for furfuryl alcohol, although at the cost of a reduced deposition rate.

EDS analysis confirmed that the obtained coatings consisted predominantly of copper, while differences between samples were mainly related to oxygen and sulfur contents associated with plating bath components and additives. The highest chemical purity was observed for layers deposited from baths containing furfural and furfuryl alcohol, whereas the use of SDS resulted in a higher fraction of non-metallic elements due to the presence of sulfate surfactant.

An optimal thickener content of 5 wt.% was identified, providing the highest tensile strength while maintaining good process stability in the electrolyte solution environment. Increasing the thickener concentration reduced stiffness (Young’s modulus), indicating a trade-off between strength and rigidity that must be considered when designing photocurable compositions for hybrid 3D printing.

Overall, the results indicate that resin compositions containing approximately 5 wt.% nanosilica are optimal for photopolymerization in electrolyte solution environments, while among the analyzed electrolyte solutions, the bath containing SDS provides the best compromise between deposition rate and structural quality. The obtained findings provide a basis for further research focused on the full integration of photopolymerization and electrodeposition processes within a single 3D printing system.

## Figures and Tables

**Table 1:** Compositions of electroplating baths used for producing electrodeposited copper layers.

No.	CuSO_4_ Concentration	pH Regulator/Supporting Electrolyte		Brightening Agent	
		Name	C [mol/dm^3^]	Name	C [mol/dm^3^]
1	saturated	H_2_SO_4_	1	-	-
2	saturated	H_2_SO_4_	1	SDS	0.010
3	saturated	Na_2_SO_4_	1	Furfural	0.010
4	saturated	Na_2_SO_4_	1	Furfuryl alcohol	0.010

**Table 2:** Summary of masses and dimensions of 3D-printed test samples.

Sample Name	Theoretical Weight [g]	Sample Weight [g]	Theoretical Dimensions [mm]	Sample Dimensions [mm]			
				a	b_1_	b_2_	h
ABS-Like 3%	1.4760	1.3155	a = 60.00 b1 = 20.00 b2 = 10.00 h = 1.50	58.32	19.27	9.72	1.21
ABS-Like 5%		1.3231		58.69	19.39	9.77	1.30
ABS-Like 7%		1.2932		57.12	19.92	9.82	1.32
Tough 3%	1.4145	1.3123		58.42	19.32	9.73	1.28
Tough 5%		1.3268		58.65	19.43	9.83	1.38
Tough 7%		1.2459		58.11	19.21	9.76	1.18

**Table 3:** Elemental composition of the analyzed electrodeposited copper layers.

Brightener	Elemental Mass Fraction [wt.%]				
	**Cu**	**O**	**S**	**Cl**	**C**
SDS	78.78	11.96	7.94	1.31	0.00
Furfuryl alcohol	90.85	4.79	4.36	0.00	0.00
Furfural	93.25	3.81	2.95	0.00	0.00
Without brightener	83.51	8.13	7.70	0.65	0.00
Cu-ETP sheet (CW004A)	89.72	0.34	0.00	0.00	9.94

## Data Availability

The original contributions presented in this study are included in the article/Supplementary Material. Further inquiries can be directed to the corresponding author.

## Supplementary Materials

The following supporting information can be downloaded at: https://www.mdpi.com/article/10.3390/molecules31081316/s1, in the Supplementary Materials, the viscosity as a function of shear rate is presented for all analyzed photocurable compositions, as well as microscopic images and EDS spectra of all electrochemically deposited copper layers and compositions of electroplating baths used in linear voltammetry tests. Figure S1. Plot of viscosity versus shear rate for the ABS-Like 0% sample, with the region used to calculate the average viscosity of the composition marked. Figure S2. Plot of viscosity versus shear rate for the Tough 0% sample, with the region used to calculate the average viscosity of the composition marked. Figure S3. Plot of viscosity versus shear rate for the ABS-Like 3% sample, with the region used to calculate the average viscosity of the composition marked. Figure S4. Plot of viscosity versus shear rate for the Tough 3% sample, with the region used to calculate the average viscosity of the composition marked. Figure S5. Plot of viscosity versus shear rate for the ABS-Like 5% sample, with the region used to calculate the average viscosity of the composition marked. Figure S6. Plot of viscosity versus shear rate for the Tough 5% sample, with the region used to calculate the average viscosity of the composition marked. Figure S7. Plot of viscosity versus shear rate for the ABS-Like 7% sample, with the region used to calculate the average viscosity of the composition marked. Figure S8. Plot of viscosity versus shear rate for the Tough 7% sample, with the region used to calculate the average viscosity of the composition marked. Figure S9. Images of the surfaces of electrodeposited copper layers obtained using electroplating baths without brightening agent. Figure S10. Images of the surfaces of electrodeposited copper layers obtained using electroplating baths with sodium dodecyl sulfate (SDS) as a brightening agent. Figure S11. Images of the surfaces of electrodeposited copper layers obtained using electroplating baths with furfural as a brightening agent. Figure S12. Images of the surfaces of electrodeposited copper layers obtained using electroplating baths with furfuryl alcohol as a brightening agent. Figure S13. EDS spectrum of the sample electrodeposited from the bath without a brightener additive. Figure S14. EDS spectrum of the sample electrodeposited from the bath containing SDS as a brightener additive. Figure S15. EDS spectrum of the sample electrodeposited from the bath containing furfuryl alcohol as a brightener additive. Figure S16. EDS spectrum of the sample electrodeposited from the bath containing furfural as a brightener additive. Table S1. Compositions of electroplating baths containing SDS used in linear voltammetry tests. Table S2. Compositions of electroplating baths containing furfural used in linear voltammetry tests. Table S3. Compositions of electroplating baths containing furfuryl alcohol used in linear voltammetry tests.

## Abbreviations

The following abbreviations are used in this manuscript:

BJ	Binder Jetting
CAD	Computer-Aided Design
DIW	Direct Ink Writing
DLP	Digital Light Processing
EBM	Electron Beam Melting
FDM	Fused Deposition Modeling
FE-SEM	Field-Emission Scanning Electron Microscope
HVAC	Heating Ventilation Air Conditioning
IV-LED	Ultraviolet Light-Emitting Diode
LMD	Laser Metal Deposition
SEM	Scanning Electron Microscopy
SDS	Sodium Dodecyl Sulfate
SLA	Stereolithography
SLM	Selective Laser Melting
SLS	Selective Laser Sintering
UV	Ultraviolet
WAAM	Wire Arc Additive Manufacturing

## References

[1] Cluff D.R.A., Esmaeili S. (2009). Compressive properties of a new metal–polymer hybrid material. J. Mater. Sci..

[2] Carradò A., Faerber J., Niemeyer S., Ziegmann G., Palkowski H. (2011). Metal/polymer/metal hybrid systems: Towards potential formability applications. Compos. Struct..

[3] Amancio-Filho S.T., Blaga L.-A. (2018). Joining of Polymer–Metal Hybrid Structures: Principles and Applications.

[4] Gomez-Romero P., Pokhriyal A., Rueda-García D., Bengoa L.N., González-Gil R.M. (2024). Hybrid Materials: A Metareview. Chem. Mater..

[5] Lambiase F., Scipioni S.I., Lee C.J., Ko D.C., Liu F. (2021). A State-of-the-Art Review on Advanced Joining Processes for Metal-Composite and Metal-Polymer Hybrid Structures. Materials.

[6] Shahrubudin N., Lee T.C., Ramlan R. (2019). An Overview on 3D Printing Technology: Technological, Materials and Applications. Procedia Manuf..

[7] Jayswal A., Adanur S. (2022). An overview of additive manufacturing methods, materials and applications for flexible structures. J. Ind. Text..

[8] Karakurt I., Lin L. (2020). 3D printing technologies: Techniques, materials and post-processing. Curr. Opin. Chem. Eng..

[9] Tomal W., Chachaj-Brekiesz A., Popielarz R., Ortyl J. (2020). Multifunctional biphenyl derivatives as photosensitisers in various types of photopolymerization processes, including IPN formation, 3D printing of photocurable multiwalled carbon nanotubes (MWCNTs) fluorescent composites. RSC Adv..

[10] Petko F., Galek M., Hola E., Topa-Skwarczyńska M., Tomal W., Jankowska M., Pilch M., Popielarz R., Graff B., Morlet-Savary F. (2022). Symmetric Iodonium Salts Based on Benzylidene as One-Component Photoinitiators for Applications in 3D Printing. Chem. Mater..

[11] Hola E., Pilch M., Ortyl J. (2020). Thioxanthone Derivatives as a New Class of Organic Photocatalysts for Photopolymerisation Processes and the 3D Printing of Photocurable Resins under Visible Light. Catalysts.

[12] Hola E., Pilch M., Galek M., Ortyl J. (2020). New versatile bimolecular photoinitiating systems based on amino-m-terphenyl derivatives for cationic, free-radical and thiol–ene photopolymerization under low intensity UV-A and visible light sources. Polym. Chem..

[13] Al Mousawi A.A., Dietlin C., Graff B., Morlet-Savary F., Toufaily J., Hamieh T., Fouassier J.P., Chachaj-Brekiesz A., Ortyl J., Lalevée J. (2016). Meta-Terphenyl Derivative/Iodonium Salt/9H-Carbazole-9-ethanol Photoinitiating Systems for Free Radical Promoted Cationic Polymerization upon Visible Lights. Macromol. Chem. Phys..

[14] Ortyl J., Popielarz R. (2012). New photoinitiators for cationic polymerization. Polimery.

[15] Topa-Skwarczyńska M., Ortyl J. (2023). Photopolymerization shrinkage: Strategies for reduction, measurement methods and future insights. Polym. Chem..

[16] Ortyl J., Wilamowski J., Milart P., Galek M., Popielarz R. (2015). Relative sensitization efficiency of fluorescent probes/sensitizers for monitoring and acceleration of cationic photopolymerization of monomers. Polym. Test..

[17] Tomal W., Krok D., Chachaj-Brekiesz A., Lepcio P., Ortyl J. (2021). Harnessing light to create functional, three-dimensional polymeric materials: Multitasking initiation systems as the critical key to success. Addit. Manuf..

[18] Trembecka-Wójciga K., Ortyl J. (2024). Enhancing 3D printed ceramic components: The function of dispersants, adhesion promoters and surface-active agents in Photopolymerization-based additive manufacturing. Adv. Colloid Interface Sci..

[19] Kristiawan R.B., Imaduddin F., Ariawan D., Arifin Z. (2021). A review on the fused deposition modeling (FDM) 3D printing: Filament processing, materials and printing parameters. Open Eng..

[20] Stansbury J.W., Idacavage M.J. (2016). 3D printing with polymers: Challenges among expanding options and opportunities. Dent. Mater..

[21] Revelo C.F., Colorado H.A. (2018). 3D printing of kaolinite clay ceramics using the Direct Ink Writing (DIW) technique. Ceram. Int..

[22] Topa M., Hola E., Galek M., Petko F., Pilch M., Popielarz R., Morlet-Savary F., Graff B., Lalevée J., Ortyl J. (2020). One-component cationic photoinitiators based on coumarin scaffold iodonium salts as highly sensitive photoacid generators for 3D printing IPN photopolymers under visible LED sources. Polym. Chem..

[23] Tomal W., Pilch M., Chachaj-Brekiesz A., Ortyl J. (2019). Development of New High-Performance Biphenyl and Terphenyl Derivatives as Versatile Photoredox Photoinitiating Systems and Their Applications in 3D Printing Photopolymerization Processes. Catalysts.

[24] Więcław-Midor A., Tańska J., Falkowski P., Wiecińska P. (2023). Photochemically induced polymerization in additive manufacturing methods for forming ceramic components. Szkło Ceram..

[25] Fina F., Goyanes A., Gaisford S., Basit A.W. (2017). Selective laser sintering (SLS) 3D printing of medicines. Int. J. Pharm..

[26] Rahman Z., Charoo N.A., Kuttolamadom M., Asadi A., Khan M.A. (2020). Printing of personalized medication using binder jetting 3D printer. Precision Medicine for Investigators, Practitioners and Providers.

[27] Murr L.E. (2018). A Metallographic Review of 3D Printing/Additive Manufacturing of Metal and Alloy Products and Components. Metallogr. Microstruct. Anal..

[28] Gadagi B., Lekurwale R. (2021). A review on advances in 3D metal printing. Mater. Today Proc..

[29] Chua K., Khan I., Malhotra R., Zhu D. (2021). Additive manufacturing and 3D printing of metallic biomaterials. Eng. Regen..

[30] Seol S.K., Kim D., Lee S., Kim J.H., Chang W.S., Kim J.T. (2015). Electrodeposition-based 3D Printing of Metallic Microarchitectures with Controlled Internal Structures. Small.

[31] Pączkowski J. (2003). Photochemistry of Polymers: Theory and Applications.

[32] Kostrzewska K., Ortyl J., Dobosza R., Kabatc J. (2017). Squarylium dye and onium salts as highly sensitive photoradical generators for blue light. Polym. Chem..

[33] Kamińska I., Ortyl J., Popielarz R. (2016). Mechanism of interaction of coumarin-based fluorescent molecular probes with polymerizing medium during free radical polymerization of a monomer. Polym. Test..

[34] Landolt D. (2002). Electrodeposition Science and Technology in the Last Quarter of the Twentieth Century. J. Electrochem. Soc..

[35] Ngo T.D., Kashani A., Imbalzano G., Nguyen K.T.Q., Hui D. (2018). Additive manufacturing (3D printing): A review of materials, methods, applications and challenges. Compos. Part B Eng..

[36] Gibson I., Rosen D.W., Stucker B. (2015). Additive Manufacturing Technologies: 3D Printing, Rapid Prototyping and Direct Digital Manufacturing.

[37] Caminero M.A., Chacón J.M., García-Moreno I., Reverte J.M. (2018). Interlaminar bonding performance of 3D printed continuous fibre reinforced thermoplastic composites using fused deposition modelling. Polym. Test..

[38] Gu D., Meiners W., Wissenbach K., Poprawe R. (2012). Laser additive manufacturing of metallic components: Materials, processes and mechanisms. Int. Mater. Rev..

[39] Lewis J.A. (2006). Direct ink writing of 3D functional materials. Adv. Funct. Mater..

[40] Tumbleston J.R., Shirvanyants D., Ermoshkin N., Janusziewicz R., Johnson A.R., Kelly D., Chen K., Pinschmidt R., Rolland J.P., Ermoshkin A. (2015). Continuous liquid interface production of 3D objects. Science.

[41] Ligon S.C., Liska R., Stampfl J., Gurr M., Mülhaupt R. (2017). Polymers for 3D printing and customized additive manufacturing. Chem. Rev..

[42] Ambrosi A., Pumera M. (2016). 3D-printing technologies for electrochemical applications. Chem. Soc. Rev..

[43] Yin S., Suo X., Liao H., Wang X. (2018). Cold spraying of metal–polymer composites: A review. J. Therm. Spray Technol..

[44] (2019). Plastics—Determination of Tensile Properties—Part 1: General Principles.

[45] Choi J.W., Kim H.C., Wicker R. (2011). Multi-material stereolithography. J. Mater. Process. Technol..

