# Supplementary material for: Initial Study of Feedstock Material Compositions for 3D Printing of Hybrid Metal–Polymer Components via Electrodeposition and Photopolymerization in an Electroplating Bath Environment
Source: Molecules. 2026 Apr 17;31(8):1316. doi: 10.3390/molecules31081316 (PMC13119453; doi:10.3390/molecules31081316)
Supplement: Supplementary file 1 [file molecules-31-01316-s001.zip › molecules-4214920-supplementary.pdf]

Supplementary Materials

Initial Study of Feedstock Material Compositions for 3D Printing of Hybrid Metal–Polymer Components via Electrodeposition and Photopolymerization in an Electroplating Bath Environment

Dawid Kiesiewicz [1], Karolina Syrek [2], Paweł Niezgoda [3], Szymon Żydowski [1], Sylwia Łagan [4] and Maciej Pilch [1,*]

1 Faculty of Civil Engineering, Cracow University of Technology, Warszawska 24, 31-155 Cracow, Poland
2 Faculty of Chemistry, Jagiellonian University, Gronostajowa 2, 30-387 Cracow, Poland; syrek@chemia.uj.edu.pl
3 Faculty of Chemical Engineering and Technology, Cracow University of Technology, Warszawska 24, 31-155 Cracow, Poland
4 Faculty of Mechanical Engineering, Cracow University of Technology, Warszawska 24, 31-155 Cracow, Poland; sylwia.lagan@pk.edu.pl
* Correspondence: maciej.pilch@pk.edu.pl; Tel.: +48-12-628-35-70

The viscosity as a function of shear rate for the ABS-like and Tough photocurable compositions with varying thickener content is presented in Figures S1–S8.

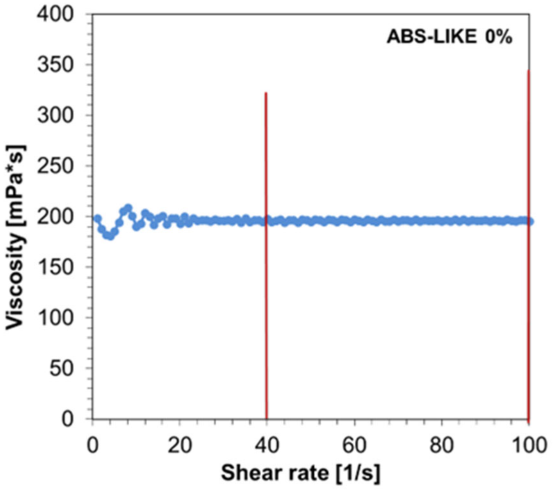

Figure S1. Plot of viscosity versus shear rate for the ABS-Like 0% sample, with the region used to calculate the average viscosity of the composition marked.

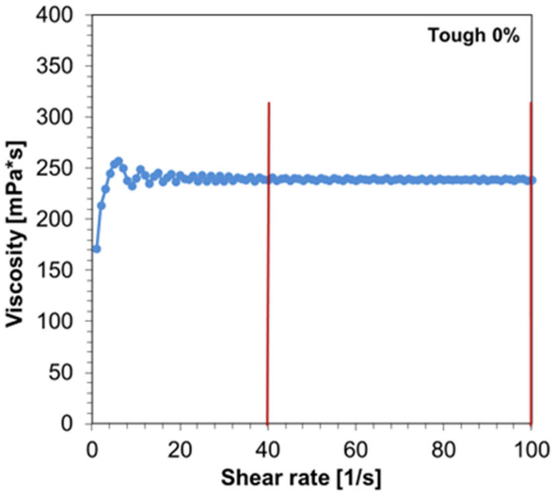

Figure S2. Plot of viscosity versus shear rate for the Tough 0% sample, with the region used to calculate the average viscosity of the composition marked.

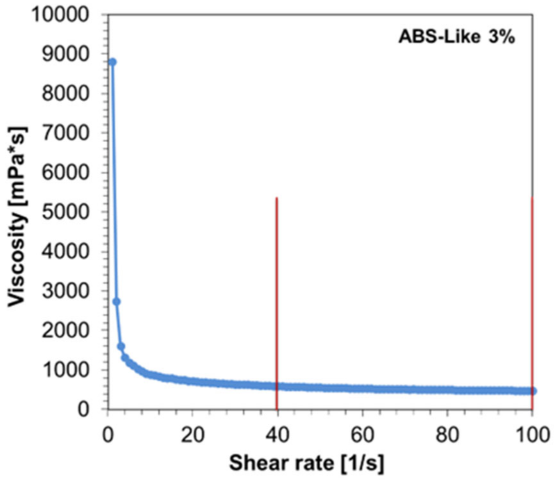

Figure S3. Plot of viscosity versus shear rate for the ABS-Like 3% sample, with the region used to calculate the average viscosity of the composition marked.

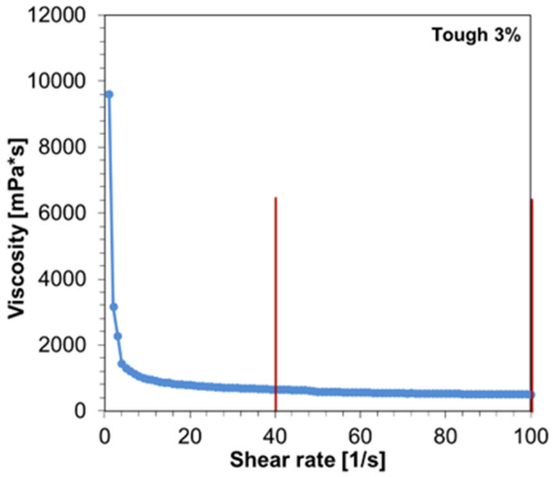

Figure S4. Plot of viscosity versus shear rate for the Tough 3% sample, with the region used to calculate the average viscosity of the composition marked.

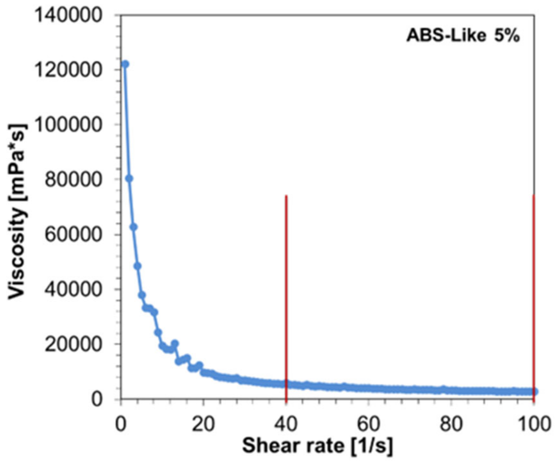

Figure S5. Plot of viscosity versus shear rate for the ABS-Like 5% sample, with the region used to calculate the average viscosity of the composition marked.

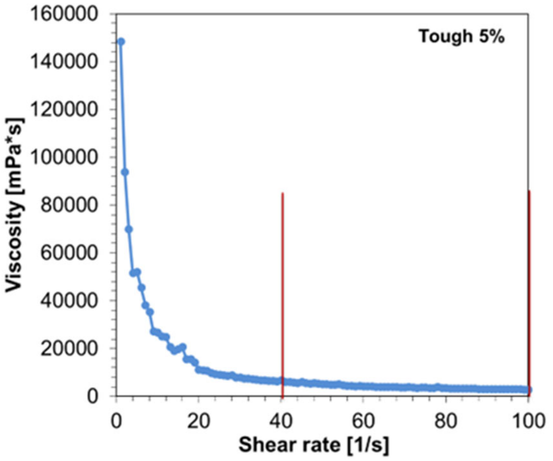

Figure S6. Plot of viscosity versus shear rate for the Tough 5% sample, with the region used to calculate the average viscosity of the composition marked.

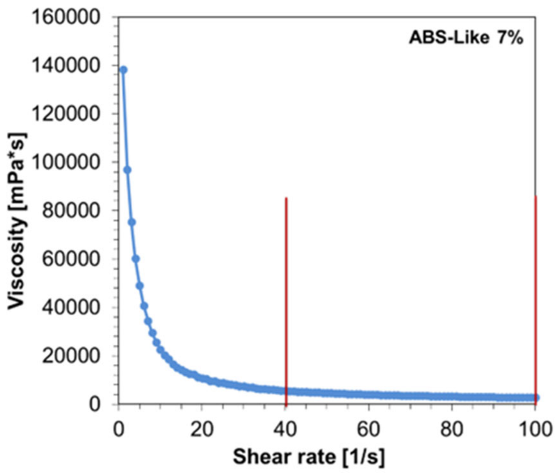

Figure S7. Plot of viscosity versus shear rate for the ABS-Like 7% sample, with the region used to calculate the average viscosity of the composition marked.

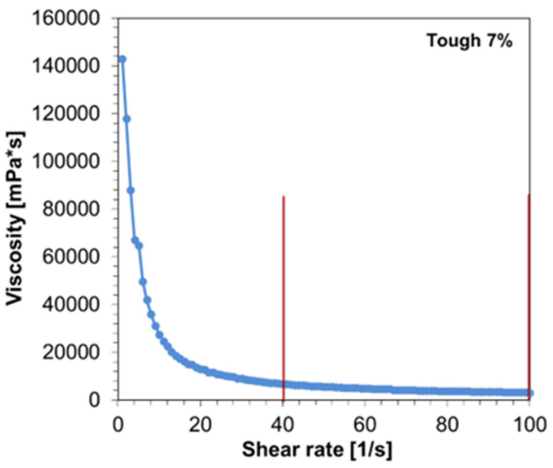

Figure S8. Plot of viscosity versus shear rate for the Tough 7% sample, with the region used to calculate the average viscosity of the composition marked.

Microscope photos of the electrodeposited copper layers are presented in Figures S9-S12. The photographs were acquired using an OLYMPUS DSX1000 optical microscope.

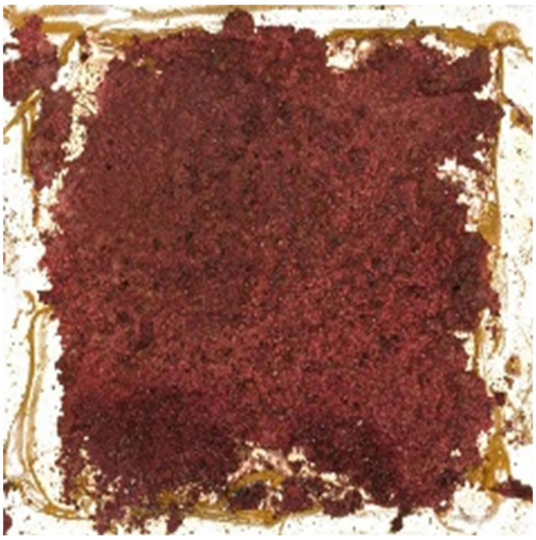

Figure S9. Images of the surfaces of electrodeposited copper layers obtained using electroplating baths without brightening agent.

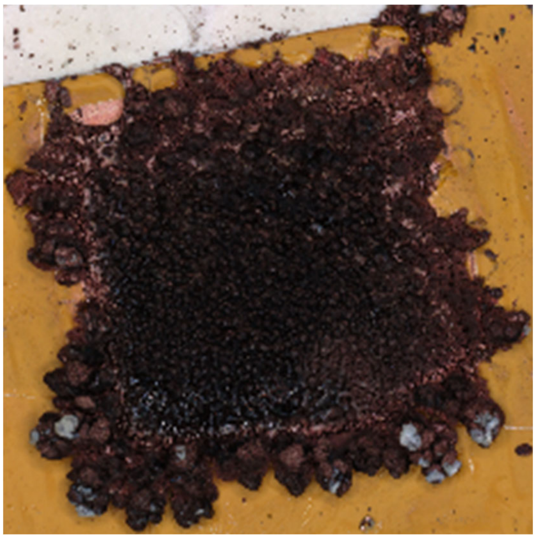

Figure S10. Images of the surfaces of electrodeposited copper layers obtained using electroplating baths with sodium dodecyl sulfate (SDS) as a brightening agent.

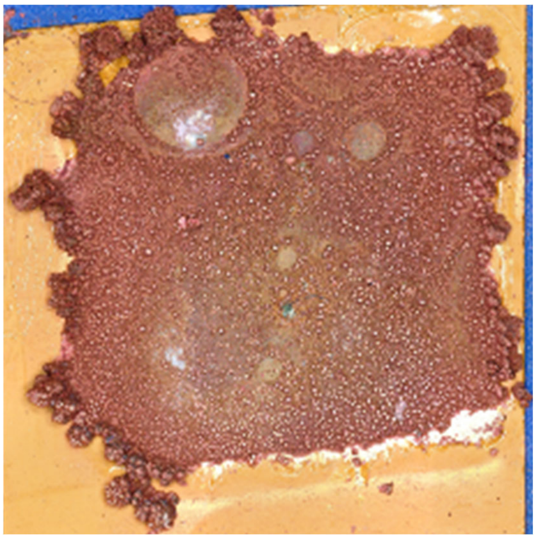

Figure S11. Images of the surfaces of electrodeposited copper layers obtained using electroplating baths with furfural as a brightening agent.

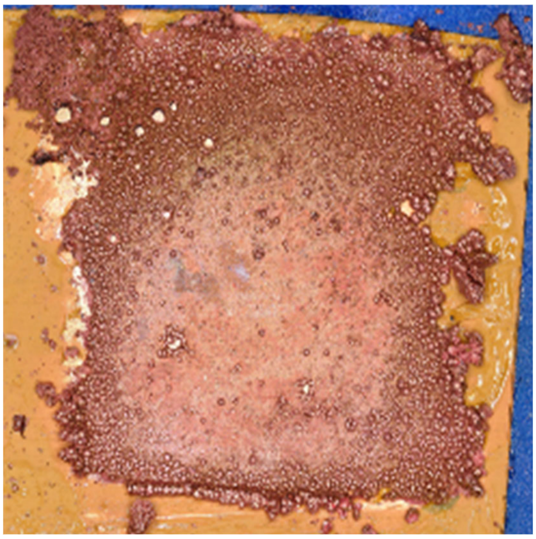

Figure S12. Images of the surfaces of electrodeposited copper layers obtained using electroplating baths with furfuryl alcohol as a brightening agent.

EDS spectra for copper samples electrodeposited from different electroplating baths are presented in Figures S13–S16.

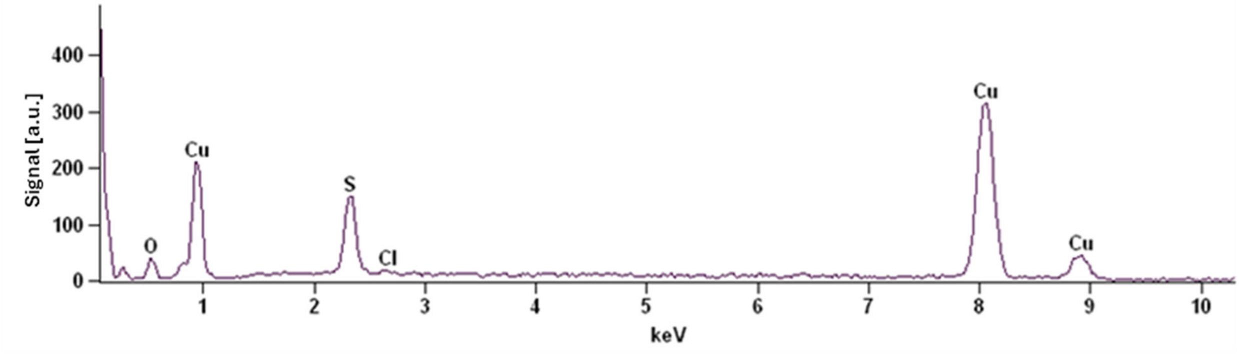

Figure S13. EDS spectrum of the sample electrodeposited from the bath without a brightener additive.

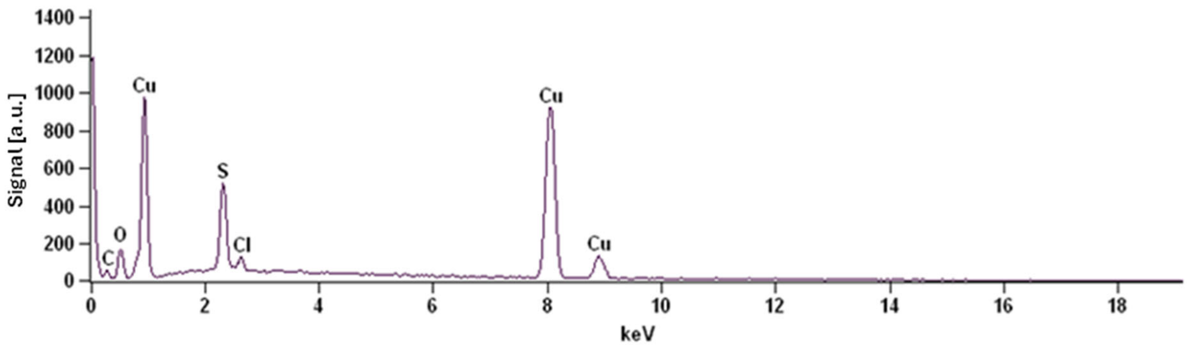

Figure S14. EDS spectrum of the sample electrodeposited from the bath containing SDS as a brightener additive.

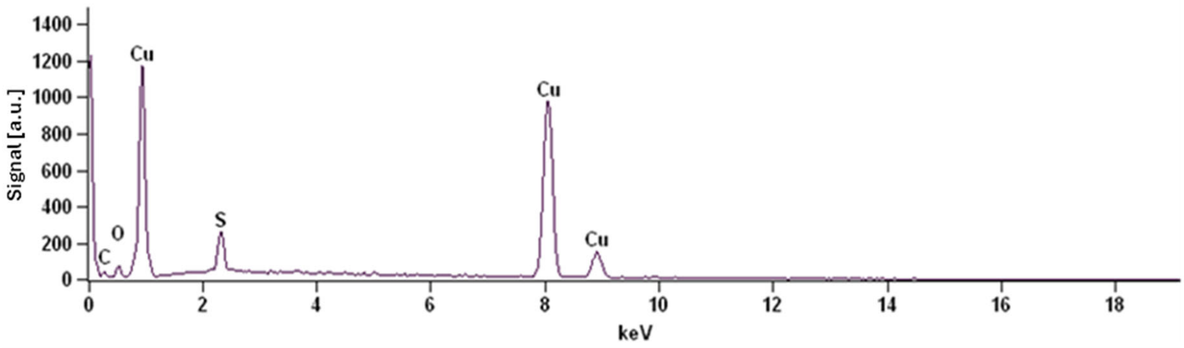

Figure S15. EDS spectrum of the sample electrodeposited from the bath containing furfuryl alcohol as a brightener additive.

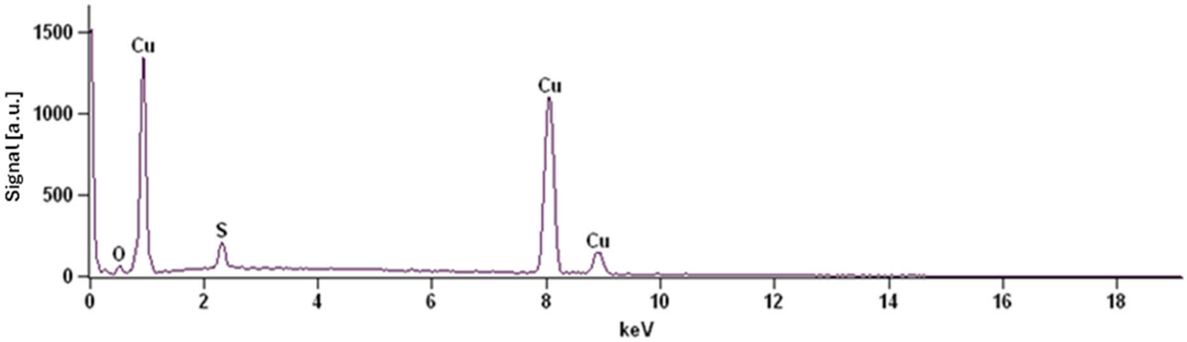

Figure S16. EDS spectrum of the sample electrodeposited from the bath containing furfural as a brightener additive.

The compositions of the investigated electroplating baths are summarized in Tables S1-S3.

Table S1. Compositions of electroplating baths containing SDS used in linear voltammetry tests.

**Sample no.**	**$CuSO_4$**	**H_2SO_4 [mol/l]**	**Brightener concentration [mmol/l]**
1			0.0
2			0.1
3			0.2
4			0.3
5			0.4
6			0.6
7	saturated	1	0.8
8			1.0
9			2.0
10			4.0
11			6.0
12			8.0
13			10.0

Table S2. Compositions of electroplating baths containing furfural used in linear voltammetry tests.

Sample no.	$CuSO_4$	Na_2SO_4 [mol/l]	Brightener concentration [mol/l]
1	saturated	1	0.00
2			0.02
3			0.04
4			0.06
5			0.08
6			0.10
7			0.20
8			0.40
9			0.60
10			0.80
11			1.00

Table S3. Compositions of electroplating baths containing furfuryl alcohol used in linear voltammetry tests.

Sample no.	$CuSO_4$	Na_2SO_4 [mol/l]	Brightener concentration [mol/l]
1	saturated	1	0.00
2			0.02
3			0.04
4			0.06
5			0.08
6			0.10
7			0.20
8			0.40
9			0.60
10			0.80
11			1.00
